# Caregiver Quality of Life: Satisfaction and Burnout

**DOI:** 10.3390/ijerph20166577

**Published:** 2023-08-14

**Authors:** Carolina Blom, Ana Reis, Leonor Lencastre

**Affiliations:** 1 CPUP—Center for Psychology at the University of Porto, Faculty of Psychology and Educational Sciences, University of Porto, 4200-135 Porto, Portugal; 2 Centro Hospitalar Universitário de São João, 4200-319 Porto, Portugal; 3 CINTESIS—Center for Health Technology and Services Research, 4200-450 Porto, Portugal

**Keywords:** cancer, informal caregivers, quality of life, burnout, caregiving satisfaction

## Abstract

Informal caregivers (ICs) of cancer patients play a crucial role in health care. Several of the challenges they face can affect their quality of life (QoL). This cross-sectional study explored role of burnout and caregiving satisfaction in their relationship to QoL. Portuguese ICs of adult cancer patients (*N* = 92) answered a sociodemographic and caregiving questionnaire, the WHOQOL-SRPB BREF, assessing physical, psychological, social, environmental, and spiritual QoL domains; the Maslach Burnout Interview, assessing the dimensions of depersonalization, emotional exhaustion, and personal accomplishment; and a Visual Analogic Scale on caregiving satisfaction. We tested correlations and a parallel mediation model for each domain of QoL, considering burnout dimensions as possible mediators between satisfaction and QoL domains. Our results show that satisfaction, burnout dimensions, and almost all QoL domains are correlated. Together, burnout dimensions seem to mediate the relationship between caregiving satisfaction and psychological, environmental, and spiritual QoL. Satisfaction had a significant indirect effect solely through emotional exhaustion on psychological QoL (β = 1.615, 95% BCI [0.590; 2.849]), environmental QoL (β = 0.904, 95% BCI [0.164; 1.876]), and spiritual QoL (β = 0.816, 95% BCI [0.019; 1.792]). It seems essential for mental health professionals to address these dimensions when providing support to an IC.

## 1. Introduction

Cancer diagnoses tend to rise by 3% yearly in Portugal [1,2]. Cancer patients undergo treatment and medical surveillance for months or years, facing difficulties in their day-to-day life [3,4]. Therefore, they need assistance for their daily living which is mostly provided by informal caregivers (ICs), usually family members, partners, or friends, who support the patient by providing unpaid care [3,5]. This support with basic and advanced activities of daily living and disease management can demand various efforts from ICs and impact several dimensions of their lives, e.g., physical, psychological, and social [5,6,7,8]. Moreover, how the caregiving experience is lived and its impact can differ according to context because of the available support services, cultural values, and role expectations about ICs [9,10,11].

Quality of life (QoL) is widely used to assess caregiving impact, e.g., [6,12,13,14]. Although it has several definitions, we adopt the proposal of the World Health Organization (WHO), where QoL is defined as “individuals’ perceptions of their position in life in the context of the culture and value systems in which they live and in relation to their goals, expectations, standards and concerns“ [15] (p. 11). According to the WHO [15], QoL incorporates the physical, psychological, social, environmental, and spiritual domains of one’s life. This definition and its measures allow for the comparison of ICs’ QoL with that of other groups and cultures [16,17]. Furthermore, it covers spiritual QoL, a dimension that might be of interest in informal caregiving, since spirituality itself seems to empower ICs and contribute to their mental health [18,19]. The systematic review by Ochoa et al. [6] shows that ICs’ QoL is low across all stages of the development of cancer and can be affected by various personal characteristics (e.g., physical health, mental health, age, sex), characteristics of the patient (e.g., autonomy and caregiving circumstances), the environment (e.g., social support, cultural environment, support policies), as well as the appraisal the caregiver makes of the situation.

Caregiving appraisal is a subjective, cognitive, and affective evaluation of potential caregiving stressors and the efficacy of the caregiver’s coping mechanisms, which can be positive, neutral, or negative [20]. Caregiving appraisal seems to have an impact not only on the IC, but also on the recipient of the care [21]. The dimension of caregiving burden, a negative form of appraisal, is very popular in the caregiving literature, but it has been criticized for its inconsistent definitions and uses [20,21,22,23]. Conversely, caregiving satisfaction, a form of positive appraisal, has been assessed to a lesser extent [20,23,24]. According to Lawton and collaborators [20], it encompasses personal satisfaction with the behaviors and emotions associated with being a caregiver and is associated with the accumulation of events that induce pleasure, affirmation, or joy. In their study of spousal caregivers of prostate cancer patients, Harden et al. [25] verified that positive appraisal in the form of the perceived benefit of caregiving had a positive effect on QoL. Studies with ICs of patients with other diseases showed that caregiving satisfaction is associated with better IC mental health and the adoption of beneficial health behaviors [26].

Given the demanding characteristics of caregiving, Gérain and Zech [22] proposed in their Informal Caregiving Integrative Model (ICIM) that burnout, a concept usually applied to work contexts, should be researched as a potential consequence of care provision. Burnout can be defined as “a psychological syndrome of emotional exhaustion, depersonalization and reduced personal fulfillment” [27] (p. 192). Gérain and Zech [22] suggested that, for ICs, emotional exhaustion refers to a situation in which the IC feels unable to continue and is emotionally drained; depersonalization refers to disconnected responses in the caregiving relationship that can lead to the objectification of the person being cared for; and the reduced personal accomplishment refers to the lack of a positive experience in fulfilling the role. The ICIM conceptualizes burnout as a “(…) consequence of the different sets of determinants, either directly or through the mediation of the appraisal and the relationship quality with the care-recipient. [Simultaneously,] caregiver burnout is also viewed as a key mediator between demands and various more general outcomes” [22] (p. 9).

Factors that may contribute to burnout can vary across cultures [11]. Research on ICs of patients with different types of diseases showed that burnout is related to lower subjective health, social support, anxiety and depression, and lower QoL in all dimensions [28,29,30,31]. The scores for emotional exhaustion and personal accomplishment in the informal caregiving literature stand out in comparison with the scores of depersonalization [31,32]. Burnout is also related to negative consequences for the care recipient. Emotional exhaustion is the sole dimension identified as a risk factor for ICs perpetrating physical violence, even when controlling for IC and patient sociodemographic variables, and received violence from the patient [31].

Considering the dimension of cancer diagnosis, and, consequently, the increase in the number of the ICs of cancer patients, it is fundamental to offer them adequate support. Furthermore, there is still little research on burnout in informal cancer-caregiving and positive appraisals of the caregiving situation. Hence, this study aimed to explore the role of burnout dimensions and caregiving satisfaction in relation to QoL domains.

## 2. Materials and Methods

### 2.1. Participants

To be part of this study, participants had to be Portuguese ICs of an adult (≥18 years) cancer patient, living in Portugal, of legal age (≥18 years), and capable of giving consent to participate. ICs suffering from impairing conditions (e.g., cancer or dementia) were asked to abstain from participating. Ninety-two ICs took part in this study.

### 2.2. Instruments

Participants filled out a sociodemographic questionnaire containing 10 questions about, e.g., IC’s age, education, work status, and a questionnaire about the caregiving situation with 17 questions about sociodemographic data of the cared-for person, data related to cancer diagnosis, caregiving duration, and weekly intensity of the caregiving role.

The WHOQOL-SRBP-BREF, developed by Skevington et al. [16] and adapted to Portuguese by Catré et al. [17], was used to assess QoL. This self-reported instrument aims to evaluate QoL as defined by the WHO in the physical, psychological, social, environmental, and spiritual domains and for overall QoL for the previous two weeks. The 34 items have to be answered on a 5-point Likert scale about capacity, frequency, intensity, or satisfaction, ranging from, for example, 1 = ”very unsatisfied” to 5 = ”very satisfied”. Each domain is scored independently. Higher scores indicate better QoL. According to Catré et al. [17], the authors of the European Portuguese version, the instrument’s internal consistency for all five domains, as measured by Cronbach’s alpha, range from acceptable (α = 0.71) to very good (α = 0.87). In this study, they ranged from acceptable (α = 0.69) to very good (α = 0.89).

The Maslach Burnout Interview-Human Services Survey, developed by Maslach et al. [27] and its Portuguese version by Melo et al. [33], aims to assess occupational burnout in people who provide health services. The instrument consists of 22 items divided into three dimensions: emotional exhaustion, depersonalization, and personal fulfillment. Each item is scored on a 7-point Likert scale, where 0 = “never” and 6 = “every day”. Results for each dimension are calculated independently. Higher values of depersonalization and emotional exhaustion and lower values of personal fulfillment are associated with burnout. In the validation study, each scale showed a Cronbach’s alpha ranging from good (α = 0.70) to very good (α = 0.80), suggesting a good internal consistency [33]. In our study, Cronbach’s alpha ranged from conditional (α = 0.60) to excellent (α = 0.90).

Caregiving satisfaction was analyzed using one item with the instruction “position yourself on the following scale, taking into account the level of satisfaction you feel about the care you provide to the patient” to be answered on a Visual Analogic Scale with two anchors at each end. The left end was identified as “not satisfied at all” and the right end as “totally satisfied”.

### 2.3. Study Design and Procedure

This study is part of a larger study on the QoL of ICs of Portuguese cancer patients. A descriptive cross-sectional design was adopted [34]. Official Portuguese Statistics in 2019 affirm there were 1059012 ICs in Portugal, but the specific numbers according to patients’ disease are still unknown [35]. Therefore, the convenience snowball sampling method was adopted. ICs were recruited online on caregiving social network groups. There was no a priori sample-size determination. We reached out to 68 representatives of Web-based groups related to cancer. Twenty-three replied and allowed the disclosure of the study in their groups. The study advertisement included a text explaining the purpose of the study and its inclusion criteria. After clicking on the advertised study’s link, ICs who gave their informed consent were directed to answer the online study protocol. Response time was between 35 and 60 min.

Data were collected using Qualtrics hosted by the University of Porto from December 2021 to October 2022. All available cancer ICs who met the inclusion criteria were included in the sample (*N* = 92). No further responses were collected due to the project’s timing and resources. A response rate of 34.20% was obtained.

**Figure 1:**
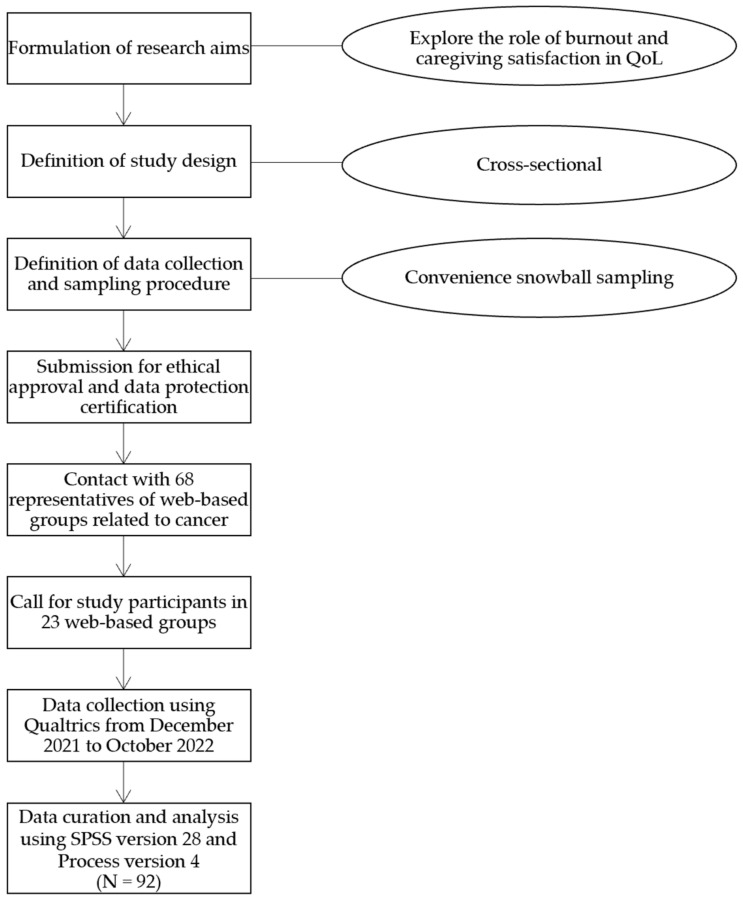
Flowchart of the overall study methodology.

The study was implemented according to the principles of the Declaration of Helsinki [36] and the Portuguese Psychologists Association [37]. Ethics approval was granted by the Faculty of Psychology and Education Sciences of the University of Porto (ref 2021/09-09b). This research was adapted to comply with European Data Protection Guidelines [38]. Data protection certification was conceded by the Data Protection Office of the University of Porto (A-15/2022). Figure 1 shows a flowchart of the overall study methodology.

### 2.4. Data Analysis

**Figure 2:**
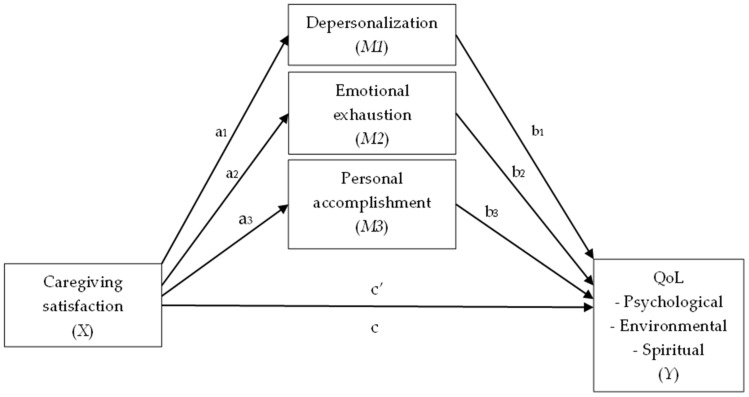
Parallel mediation models.

Data were analyzed using IBM SPSS (version 28, New York, United States) and the PROCESSmacro (version 4.2) [39]. There were no missing data. Descriptives and Pearson’s correlations between study variables were run. Our hypothesis that emotional exhaustion, depersonalization, and personal accomplishment could interfere in the relationship between caregiving satisfaction and QoL was tested using a parallel mediation model, where our predictor variable (X) was caregiving satisfaction, all our mediators (M_1,2,3_) were a single dimension of burnout, and our outcome variables (Y) were the QoL domains (see Figure 2). The use of parallel mediation seemed adequate, since the three dimensions of burnout are correlated, but not expected to casually influence each other [39]. Also, we did not know if each burnout dimension plays a different role in the mediation between caregiving satisfaction and QoL when the three are considered altogether. Before performing the latter analysis, simple and multiple regression models were tested for each outcome variable: X predicting Y; X predicting M_1_; X predicting M_2_; X predicting M_3_; M_1_ predicting Y; M_2_ predicting Y; M_3_ predicting Y; and X and M_1,2,3_ altogether predicting Y. Tolerance and variance inflation factors were analyzed to assess multicollinearity. Normal probability plots and scatterplots were analyzed to understand normality of estimation errors, homoscedasticity, and linearity. Durbin–Watson statistics were computed to determine the independence of errors in the model. These analyses were performed to assess conditions and fulfillment of assumptions for mediation [39,40].

Overall QoL and physical QoL did not correlate significantly with caregiving satisfaction, and social QoL did not correlate significantly with depersonalization. Additionally, social QoL and satisfaction with caregiving showed non-independent errors (Durbin–Watson’s d = 1.44). Therefore, these variables were not used in mediation models. In the end, three parallel mediation models were analyzed for each one of the QoL domains. Parallel mediation was tested using model 4 in the Process macro. The parallel mediation estimated direct and indirect effects based on 95% bias-corrected confidence interval using 5000 bootstrap samples [39].

Following the recommendations from Schoemann et al. [41], a posteriori power analysis was conducted for correlation of the bivariate normal model using G*Power (version 3.1.) [42]. Considering our sample size (*N* = 92), a significance level (α) of 0.05, and correlations ranging between r = 0.3 and r = 0.6, the obtained statistical power was (1−β) ≥ 0.90.

## 3. Results

### 3.1. Participants

Participants’ characteristics can be seen in Table 1. Ninety-two ICs who were, on average, 40 years old (*SD* = 10.87) took part in this study. Most of them were female (89.1%) and daughters/sons (48.9%) or spouses/partners (38%) of the cancer patient and living with them (72.8%). Cancer patients were, on average, 63.67 years old (*SD* = 13.09) and mostly male (57.6%). In Table 2, we can see that lung (12.4%), colon (10.1%), breast (9%), and multiple myeloma (9%) were the most frequent types of cancer diagnoses. On average, ICs reported providing care for about 22 months (*SD* = 27.26) and 11.21 h per day (*SD* = 8.78).

### 3.2. Relationship between Caregiving Satisfaction, Burnout, and QoL

Descriptives and correlations between the study variables can be seen in Table 3. The mean caregiving satisfaction score was 7.79 (*SD* = 2.23), suggesting most caregivers were satisfied with the care they provided for the patients. Depersonalization levels were, on average, 4.61 (*SD* = 4.81); those for emotional exhaustion were 23.56 (*SD* = 13.71); and those for personal accomplishment were 36.04 (*SD* = 8.74). As for QoL domains, on average, participants scored highest on spiritual QoL (*M* = 60.96; *SD* = 19.61) and lowest on social QoL (*M* = 51.45; *SD* = 21.63).

Correlations between caregiving satisfaction and all dimensions of burnout were moderate (Person’s R ranging between |0.39| and |0.51|) and significant. The relationships between caregiving satisfaction with emotional exhaustion and depersonalization were negative, and this was positive with personal accomplishment. Correlations between caregiving satisfaction and QoL domains were positive, but small (Person’s R ranging between 0.19 and 0.33) and significant for all dimensions, except for overall QoL and physical QoL. The correlation of burnout dimensions with QoL domains were small to moderate and significant (Person’s R ranging between |0.22| and |0.61|), except for social QoL with depersonalization and emotional exhaustion with overall QoL.

### 3.3. Mediation Results

All results from the mediation analysis can be seen in Table 4. For all three parallel mediation models, results based on 5000 bootstrapped samples with a 95% CI indicated that the total effect of caregiving satisfaction on psychological (β_c_= 1.970, *p* = 0.014), environmental (β_c_= 2.554, *p* = 0.0042), and spiritual QoL (β_c_= 2.361, *p* = 0.01) was significant, but the direct effect was not (β_c′_= 0.111, *p* = 0.888; β_c′_= 1.047, *p* = 0.226; and β_c′_= 1.021, *p* = 0.322, respectively). The three burnout dimensions together showed an indirect effect on the relationship between caregiving satisfaction and psychological QoL (β_ab_ = 1.859, 95% BCI [0.738; 3.110]), environmental QoL (β_ab_ = 1.507, 95% BCI [0.504; 2.682]), and spiritual QoL (β_ab_ = 1.340, 95% BCI [0.170; 2.807]). These results suggest that caregivers who felt more emotional exhaustion and depersonalization and less personal accomplishment had lower levels of psychological, environmental, and spiritual QoL.

Emotional exhaustion was the only mediator which significantly contributed to the indirect effect on psychological QoL (β_a2b2_ = 1.615, 95% BCI [0.590; 2.849]), environmental QoL (β_a2b2_ = 0.904, 95% BCI [0.164; 1.876]), and spiritual QoL (β_a2b2_ = 0.816, 95% BCI [0.019; 1.792]). Therefore, caregivers who reported higher emotional exhaustion were more likely to report lower scores in these domains.

## 4. Discussion

This study aimed to explore the role of burnout and caregiving satisfaction in relation to QoL. Our results show caregiving satisfaction, depersonalization, personal accomplishment, and almost all QoL domains were correlated (except for social QoL and depersonalization). Together, emotional exhaustion, depersonalization, and personal accomplishment mediated the relationship between caregiving satisfaction and psychological, environmental, and spiritual QoL. A significant effect was attributable to solely emotional exhaustion. Little is known about burnout and caregiving satisfaction in the ICs of cancer patients, but studies with other populations and theoretical models point out possible relationships between these concepts [20,22,28].

Participant sociodemographic characteristics of ICs and patients in this study were similar to the ones from previous studies [3,6]. Most of the participants were female and the spouse/partner of the cancer patients, and the age range were very wide, from young adulthood to older adults. Lung, colon, breast, and prostate cancer, the most common types of cancer in Portugal, were represented in the cancer patients of the ICs participating in this study [1].

As expected, QoL scores were lower than the results of a sample of the general Portuguese population [6,17]. Although the highest scores on QoL domains for the Portuguese population were verified in physical QoL, in our study, spiritual QoL scored highest. Spirituality is portrayed in the literature as a resource for coping with disease, which could explain the higher values of spiritual QoL compared with other dimensions [18]. Physical QoL was the second highest in our study and the only domain to which caregiving satisfaction was not significantly related. One possibility that could explain this result is Portuguese society’s larger focus on physical health and easier access to physical health care services compared with other types of support [43]. Curiously, the physical dimension of QoL was also unrelated to caregiving satisfaction in the study by Harden et al. [25].

Results for caregiving satisfaction showed that ICs were mostly satisfied with the care they were providing. Prieto et al.’s study [44] also verified high levels of caregiving satisfaction in ICs of children with medical complexities, as opposed to the study from Fekete et al. [26], where ICs of patients with spinal cord injuries were, for the most part, dissatisfied with caregiving. Following the rationale of Lawton et al. [20], caregiving appraisal conceptualization, and evidence from other studies, it was no surprise that caregiving satisfaction was positively related to QoL domains and negatively related to depersonalization and emotional exhaustion [26,45,46].

In relation to the cut-off points used by Viana et al. [47] in their study with Portuguese formal caregivers, the mean score of depersonalization, emotional exhaustion, and personal accomplishment in our sample can be labeled as “medium”. Of all burnout dimensions, personal accomplishment scored highest and depersonalization scored lowest in our study. This trend also happened in other studies of ICs [29,31,47]. The burnout results in our study were similar to the ones in Truzzi et al.’s study [28] with ICs of cancer patients, but higher for emotional exhaustion when compared with the study by Kokurcan et al. [30] with a sample of ICs of schizophrenia patients. Perhaps this result can be interpreted considering the higher life expectancy for schizophrenia patients. Although cancer can be treated in many cases, it is viewed as a life-threatening disease since it is the second leading cause of death in Portugal [2]. This fact could add to the emotional strain on ICs of cancer patients. Additionally, the latter studies were conducted within other cultures, and cultural differences might be of importance when studying burnout [11].

ICs usually have an attachment relationship with the person they care for before the disease, which implies they have a meaningful bond with the patient. This could explain lower depersonalization results in our study and across IC research on burnout [24,28,31]. Questioning the concept of depersonalization in informal caregiving or its adaptation seems necessary [31]. The relationship type could also support the higher results on personal accomplishment, in the sense of having a positive impact on the life of a significant other, as well as alignment with a cultural value system which expects and praises informal care provision [6,10].

All burnout dimensions were significantly correlated with caregiving satisfaction and QoL domains, as expected, except for depersonalization with social QoL [10,24]. Based on the proposal of the ICIM, we tested the relationship of the three burnout dimensions with caregiving satisfaction and QoL and verified its possible mediating role [22]. This finding can be explained by the positive role caregiving satisfaction can play in attenuating deleterious effects on ICs’ mental health, thus supporting their QoL [21,26]. Emotional exhaustion stands out in the mediation models as the dimension that most significantly contributes to the indirect effect of caregiving satisfaction on psychological, environmental, and spiritual QoL.

The results of this study should be considered in light of some issues. The cross-sectional design and convenience sampling procedure, which relied on non-random selection, were shortcomings of this work. These can bias our results and prevent their generalizability to the broader population, since bootstrapping analysis should preferably be conducted in representative samples. Participant recruitment should be reinforced and conducted face-to-face to avoid missing out on ICs with difficulties accessing online groups. Nonetheless, the comparison between our study’s participants and the ones from other studies reassured us. The internal consistency levels of depersonalization in our study are weak. Considering the scarce literature on burnout, future studies with more a robust design should further examine the role of burnout in informal caregiving. In particular, they should look at the depersonalization dimension to understand its relevance for ICs and assess the possible mediation role of burnout with other independent variables and outcomes. The direct comparison of burnout and caregiving satisfaction between ICs of cancer patients and ICs of patients with other diseases would also be interesting to analyze.

## 5. Conclusions

ICs of cancer patients play a crucial role in health care and, therefore, the positive and negative consequences of caregiving should be known to provide them with adequate support. Their well-being is of public interest since the care they provide diminishes the workload of health care systems. Our results seem to indicate that caregiving satisfaction, burnout dimensions, and QoL domains are related. Also, they appear to back the ICIM in its suggestion that burnout could mediate the relationship between appraisal and the general outcomes of care provision, such as QoL. Burnout is linked to potential adverse consequences of care provision, such as IC depression, lower QoL, and the perpetration of violence towards patients, whereas caregiving satisfaction is linked to positive outcomes [25,26,28,29,30,31]. If more research supports this view, in the future, professionals working with ICs should assess burnout, paying particular attention to emotional exhaustion and caregiving satisfaction to identify ICs with difficulties in care provision and support them in maintaining or bettering their QoL. These practices should be recommended by decision-makers in guidelines for cancer care and favored by allowing longer appointment times with cancer patients and their ICs. The results can also add to the adaptation of current social policies for IC support.

## Figures and Tables

**Table 1:** Characteristics of informal caregivers, patients, and the caregiving situation.

	Range	*M* (*SD*)
Caregiver’s age	22–72	40 (10.87)
Patient’s age	22–87	63.67 (13.09)
	*n*	%
Caregiver’s sex		
Female	82	89.1
Male	10	10.9
Patient’s sex		
Female	39	42.4
Male	53	57.6
Caregiver’s education		
Less than high school	7	7.6
High school	47	51.1
Post-secondary education	38	41.3
Patient’s education		
Less than high school	55	59.8
High school	27	29.3
Post-secondary education	10	10.9
Caregiver’s work status		
Working full time	49	53.2
Working part time	8	8.7
Retired	7	7.6
Unemployed	18	19.6
On sick leave	9	9.8
Student	1	1.1
Relationship of caregiver to cancer Patient		
Daughter/son	45	48.9
Spouse/partner	35	38
Other	12	13.1
Cohabitation		
Living with the patient	67	72.8
Not living with the patient	25	27.2

Note: *N* = 92.

**Table 2:** Characteristics of the caregiving situation.

	Range	*M* (*SD)*
Duration of caregiving, in months	1–144	21.90 (27.26)
Daily hours spent caregiving	1–24	11.21 (8.78)
	*n*	%
Cancer Type		
Lung	11	12.4
Colon	9	10.1
Breast	8	9
Multiple myeloma	8	9
Prostate	6	6.7
Melanoma	4	4.5
Stomach	4	4.5
Other	42	43.8

Note: *N* = 92.

**Table 3:** Descriptive statistics, Cronbach’s alpha, and correlations for study variables.

Variable	*M (SD)*	α	Sk	Ku	1	2	3	4	5	6	7	8	9	10
1. Caregiving satisfaction	7.79 (2.23)	—	−0.95	0.05	—									
2. Depersonalization	4.61 (4.81)	0.60	0.90	0.04	−0.39 **	—								
3. Emotional exhaustion	23.56 (13.71)	0.90	0.01	−1.07	−0.38 **	0.61 **	—							
4. Personal accomplishment	36.04 (8.74)	0.82	−0.52	−0.39	0.51 **	−48 **	−0.54 **	—						
5. Physical QoL	60.29 (15.76)	0.76	−0.01	0.41	0.17	−0.31 **	−0.50 **	0.28 **	—					
6. Psychological QoL	55.00 (17.16)	0.79	0.19	−0.41	0.26 *	−0.31 **	−0.57 **	0.39 **	0.61 **	—				
7. Social QoL	51.45 (21.63)	0.72	−0.31	−0.37	0.22 *	−0.14	−0.28 **	0.22 *	0.38 **	0.48 **	—			
8. Environmental QoL	56.28 (17.45)	0.84	−0.58	0.11	0.33 **	−0.39 **	−0.46 **	0.35 **	0.58 **	61 **	0.54 **	—		
9. Spiritual QoL	60.96 (19.61)	0.89	−0.33	−0.27	0.27 **	−0.30 **	−0.37 **	0.29 **	0.36 **	0.67 **	0.46 **	0.49 **	—	
10. Overall QoL	50.68 (19.73)	0.69	−0.38	−0.24	0.18	−0.22 *	0.18	0.28 **	0.61 **	0.47 **	0.53 **	0.57 **	0.37 **	—

Note: *N* = 92; ** *p* ≤ 0.01; * *p* ≤ 0.05.

**Table 4:** Parallel mediation models.

Models and Paths	Effect	s.e.	Bootstrap 95% CI
A1	−0.832 ***	0.210 ***	—
A2	−2.341 ***	0.600 ***	—
A3	1.994 ***	0.356 ***	—
Psychological QoL model			
B1	0.339	0.404	—
B2	−0.690 ***	0.147 ***	—
B3	0.264	0.223	—
Direct effect (C′)	0.111	0.793	—
Total effect (C)	1.970 *	0.785*	—
Indirect effect via mediators	1.859	0.595	[0.738; 3.110]
Depersonalization	−0.282	0.397	[−1.133; 0.477]
Emotional exhaustion	1.615	0.579	[0.590; 2.849]
Personal accomplishment	0.526	0.785	[−0.232; 1.455]
Model R^2^	0.343 ***		
Environmental QoL model			
B1	−0.480	0.437	—
B2	−0.386 *	0.159 *	—
B3	0.102	0.242	—
Direct effect (C′)	1.047	0.860	—
Total effect (C)	2.554 **	0.780 **	—
Indirect effect via mediators	1.507	0.560	[0.504; 2.682]
Depersonalization	0.399	0.486	[−0.554; 1.413]
Emotional exhaustion	0.904	0.442	[0.164; 1.876]
Personal accomplishment	0.204	0.540	[−0.824; 1.341]
Model R^2^	0.253 ***		
Spiritual QoL model			
B1	−0.315	0.521	—
B2	−0.348	0.190	—
B3	0.132	0.288	—
Direct effect (C’)	1.021	1.025	—
Total effect (C)	2.361 **	0.893	—
Indirect effect via mediators	1.340	0.679	[0.170; 2.807]
Depersonalization	0.262	0.546	[−0.733; 1.464]
Emotional exhaustion	0.816	0.451	[0.019; 1.792]
Personal accomplishment	0.263	0.601	[−0.882; 1.532]
Model R^2^	0.160 **		

Note. *N* = 92; *Bootstrap sample size =  5000;* *** *p* ≤ 0.001; ** *p* ≤ 0.01; * *p* ≤ 0.05.

## Data Availability

Data are unavailable due to data protection restrictions.
